# Supplementary material for: Akkermansia muciniphila alleviates experimental colitis through FXR-mediated repression of unspliced XBP1
Source: mSystems. 2025 Dec 29;11(2):e01589-25. doi: 10.1128/msystems.01589-25 (PMC12911386; doi:10.1128/msystems.01589-25)
Supplement: Fig. S1 — Expression levels of ATF6. [file msystems.01589-25-s0001.docx]

**Supplementary figure**

**Figure S1**


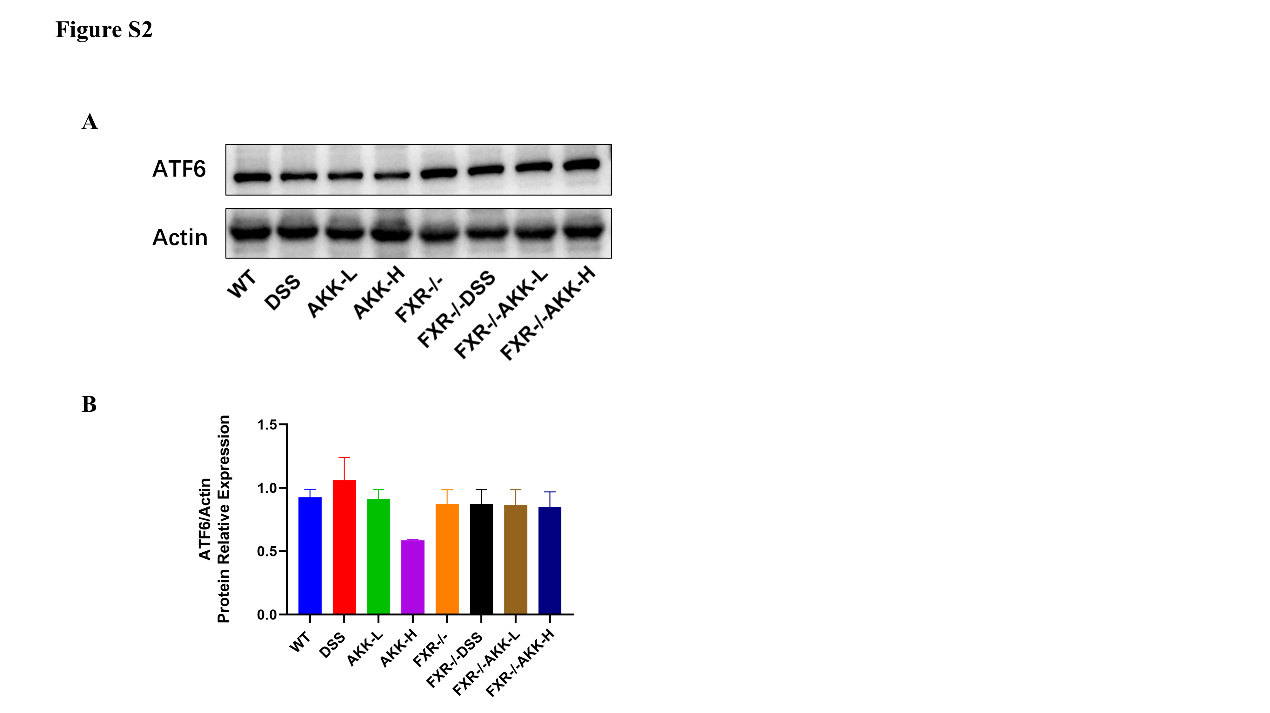


**Figure S1.** (A)Western blot was used to detect the expression levels of ATF6. (B) The relative quantitative analysis of ATF6 was performed.
